# A Conserved Glycoside Hydrolase Family 7 Cellobiohydrolase PsGH7a of *Phytophthora sojae* Is Required for Full Virulence on Soybean

**DOI:** 10.3389/fmicb.2020.01285

**Published:** 2020-07-02

**Authors:** Xinwei Tan, Yuyao Hu, Yuli Jia, Xiaoyuan Hou, Qian Xu, Chao Han, Qunqing Wang

**Affiliations:** ^1^Shandong Province Key Laboratory of Agricultural Microbiology, Department of Plant Pathology, College of Plant Protection, Shandong Agricultural University, Tai’an, China; ^2^State Key Laboratory of Crop Biology, Shandong Agricultural University, Tai’an, China

**Keywords:** *Phytophthora sojae*, glycoside hydrolase 7, virulence, soybean, cellobiohydrolase

## Abstract

Phytopathogens deploy glycoside hydrolases (GHs) to disintegrate plant cell walls for nutrition and invasion. However, the pathogenic mechanisms of the majority of GHs in virulence remain unknown, especially in oomycetes. In this study, a *Phytophthora sojae* gene encodes a GH7 family cellobiohydrolase, named *PsGH7a*, was identified. *PsGH7a* was highly induced during the cyst germination and infection stages. PsGH7a is conserved in oomycetes, and shares a high amino acid sequence identity (>85%) within *Phytophthora* genus. The recombinant PsGH7a catalyzes the hydrolysis of β-1,4-glucan and avicel, which represent the major components of cellulose in plant cell wall. The mutation of catalytic residue Glu236 to alanine resulted in a lower catalytic activity. In addition, the PsGH7a promotes *Phytophthora* invasion, while the mutant can not. Notably, PsGH7a protein triggers hypersensitive cell death in diverse plants. PsGH7a knockout mutants were generated via CRISPR/Cas9 system, to investigate its biological function. Compared to wild-type strain P6497, the mutants showed reduced virulence on susceptible soybean, indicates PsGH7a is indispensable to *P. sojae* virulence.

## Introduction

The battle between plants and microbes is the product of million-years of co-evolution. The front line of the plant defense is numerous physical barriers such as the cell walls, waxes and hairs (Hématy et al., 2009). A primary challenge for microbial pathogens is to penetrate the formidable and dynamic barrier of plant cell walls, which are constructed of cellulose, hemicellulose, pectin, and joined by complex distinct connection types (Somerville et al., 2004; Vorwerk et al., 2004; Wang et al., 2019).

Plant pathogens produce cell wall degrading enzymes (CWDEs) as part of their arsenal for nutrition and plant invasion (Faure, 2002; Martinez et al., 2004; Hashimoto et al., 2007; Hématy et al., 2009; Bakunina et al., 2013; van Wyk et al., 2017; Pluvinage et al., 2019). Phytopathogenic fungi and oomycetes are unique microbial pathogens that being able to break the intact physical surfaces of host plants (Soanes et al., 2007). Many plant-pathogenic fungi secrete a range of CWDEs to degrade the host cell wall, such as glycoside hydrolases, polysaccharide lyases, and esterases, even much more than that in *Trichoderma reesei*, which is known as a major industrial cellulase-producing fungus (Ma Y. A. et al., 2015; Gui et al., 2017; Le Mauff et al., 2019). For example, the genome of phytopathogenic fungi *Magnaporthe grisea* and *Fusarium graminearum* contains two to three times of genes encoding cellulases and xylanases as that in industrial fungus *T. reesei* (Martinez et al., 2008; King et al., 2011). The effects of the CWDEs usually support their direct contributions to invasion and disease. Emasculation of the endo-beta-1,4-xylanase xyn11A affected the virulence of *Botrytis cinerea* (Brito et al., 2006), and the mutation of pectate lyase CcpelA had a marked effect on the aggressiveness of *Colletotrichum coccodes* toward tomato fruits (Ben-Daniel et al., 2012). Oomycetes, from the kingdom Stramenopila (Baldauf, 2003; Yutin et al., 2008), encompass numerous phytopathogens such as *Phytophthora*, *Pythium*, *Albugo*, and downy mildews, which genomes encode abundant of CWDEs toward plant cell wall components (Tyler et al., 2006; Haas et al., 2009; Baxter et al., 2010). It is reported that *Phytophthora sojae*, the causal agent of soybean stem and root rot disease, manipulates plant immunity by protecting xyloglucanase XEG1 through its truncated paralogous PsXLP1 as a decoy (Ma et al., 2017). Nevertheless, the pathogenetic roles of the vast majority of CWDEs remain unknown, especially in oomycetes (Ma Z. et al., 2015).

The CWDEs produced by microorganisms were shown as multiple components, and their synergistic effect was reported (King et al., 2011; Payne et al., 2015). Some reports indicate that the glycoside hydrolases (GHs) is required for pathogen virulence on host, but the virulence mechanism are still unknown (Ma Z. et al., 2015; Baker et al., 2016; Ma et al., 2017; Fleming et al., 2017; Gui et al., 2017; Snarr et al., 2017; Le Mauff et al., 2019; Shen et al., 2020). Glycoside hydrolases are a widely distributed group of carbohydrate active enzymes (CAZy), which hydrolyze glycosidic bonds in glycosides, glycans and glycoconjugates (Henrissat and Davies, 2000). The primary action mode glycoside hydrolases is regarded as endoglucanases (EGs) act by cleaving β-1,4-glucosidic linkages in amorphous regions of cellulose chains, and cellobiohydrolases (CBHs) attach to the chain end of cello-oligosaccharides and then depolymerize these cellulosic fibers into disaccharide units (Payne et al., 2015). So far, the CAZy database^1^ classified glycoside hydrolases into 167 families based on predicted structures and sequence similarities (Turbe-Doan et al., 2019). Of these, GH7 family is somewhat enigmatic because it contains both CBHs and EGs in terms of the similar protein comformation, almost undoubtedly it provide the majority of hydrolytic turnover (Wood, 1985). This feature permits efficient hydrolysis activity, and reduces the possibly that the broken chain can reanneal into the crystal surface (Kurasin and Valjamae, 2011). In virtually, all organisms employing GH7 cellulases to degrade lignocellulosic biomass possess multiple genes of GH7 cellulases. To date, the CAZy database lists nearly 5000 GH7 sequences, including EGs and CBHs, and these GH7 cellulases offer complicated evolutionary branches with each other (Lombard et al., 2014).

In addition, unlike some prevalent GH families, such as GH5, GH6, GH12, and GH45, GH7 enzymes mainly consist in fungi, but have not been found in bacteria or archaea (Payne et al., 2015). The cellulose depolymerization of GH7 could be employed by some biomass-degrading fungi, such as the white-rot basidiomycete *Phanerochaete chrysosporium*, for nutrition and to promote entry into plant tissue (Martinez et al., 2004; Vanden Wymelenberg et al., 2006). So far, there is little research on the GH7 enzymes of phytopathogenic pathogens. Here we have isolated and characterized a gene of oomycete *P. sojae*, named *PsGH7a*, encoding a cellobiohydrolases belonging to the glycoside hydrolase family 7. *PsGH7a* is up regulated during early infection, and the protein product promotes the invasion of *Phytophthora* pathogens. The deletion of *PsGH7a* had pronounced effects on *P. sojae* virulence, delaying the rot of hypocotyls and reducing the lesion size on soybean leaves.

## Materials and Methods

### Phylogenetic Analysis of PsGH7a Homologs

All sequences of PsGH7a Homologs from oomycetes and fungi were obtained from NCBI (National Center for Biotechnology Information) website. Sequence alignments were generated via the Clustal Omega program (Sievers et al., 2011). The phylogenetic tree was constructed using MEGA 6.0 program via a neighbor joining algorithm with 1,000 bootstrap replicates.

### Plant and Phytophthora Cultivation

*Nicotiana benthamiana*, soybean (*Glycine max*), and tomato (*Solanum lycopersicum* L.) plants were grown in the chamber at 25°C with a cycle of 16 h of high light intensity and 8 h of darkness. *P. sojae* strain P6497 and *Phytophthora capsici* strain LT1534 and all transformants were grown on 10% V8 medium at 25°C in the dark. 1 × 1-mm hyphal plugs were cultured in V8 liquid medium. After 48 h, mycelia were collected for RNA and DNA extraction. For the expression pattern analysis, mycelia (MY), as well as infection stages (10 min, 30 min, 1, 3, 6, 12, and 24 h), were collected as described previously (Ye et al., 2011).

### Nucleic Acid Manipulation and Quantitative PCR Assay

The DNAMAN software was used to analyze the genes and help to design primers. The signal peptides of the proteins were predicted at the SignalP4.0 Server^2^. Genomic DNA of the *P. sojae* strains for gDNA PCR or biomass assay was isolated using HP Plant DNA kit (OMEGA Bio-Tek, Norcross, GA, United States), respectively. Total RNA of *P. sojae* was extracted using EZNA Total RNA Kit I (OMEGA Bio-Tek, Norcross, GA, United States). The concentration of DNA or RNA was measured using a spectrophotometer, and the mass was determined by agarose gel electrophoresis. cDNA was synthesized with HiScript Reverse Transcriptase Kit (Vazyme, Nanjing, China). The *PsGH7a* gene was amplified using Phanta Super-Fidelity DNA Polymerase (Vazyme, Nanjing, China) from the cDNA. The mutagenesis of PsGH7a^*E*236*A*^ was generated by using Mut Express II Fast Mutagenesis Kit V2 (Vazyme, Nanjing, China) following the previous protocol (Han et al., 2018).

Quantitative RT-PCR was performed with the *PsGH7a* forward primer TCAAGGAACCTACGGCATCAC and the reverse primer AGTTCACTCTCGACGTGGAC. The actin gene (PsActin = Ps108986) was used as an internal reference to detect *PsGH7a* transcription levels changes. Relative *P. sojae* biomass in infected plan tissue were quantified with qPCR as described previously (Wang et al., 2011). Primers used in this study are listed (Supplementary Table S2).

### Heterologous Expression and Immunoblotting

After PCR progress, the purified *PsGH7a* PCR products and pPIC9K vector were incubated with *Eco*RI and *Not*I (Fermentas, Glen Burnie, MD, United States) and ligated using T4 DNA ligase (Fermentas, Glen Burnie, MD, United States). The recombinant plasmid pPIC9K/*PsGH7a* was linearized with the restriction enzyme *Sac*I and then transformed into *Pichia pastoris* GS115. The transformants were seeded onto minimal dextrose (MD) plates and minimal methanol (MM) plates and were then screened on yeast peptone dextrose (YPD) agar medium supplemented with different concentrations of G418 (geneticin; Sangon, Shanghai, China) for the selection of multicopy integrants.

Methanol-induced enzyme expression was performed with shaking cultivation (200 rpm, 28°C) for 7 days based on the *Pichia* Expression Kit (Invitrogen, Carlsbad, CA, United States). Subsequently, the cell-free supernatant was harvested by centrifugation at 8,000 rpm for 20 min. The obtained supernatant liquor was adjusted with ammonium sulfate with 80% saturation at 4°C overnight (Han et al., 2018). Then, the precipitate was dissolved in phosphate buffer solution (PBS, 20 mM, pH 7.4). After dialysis with PBS, the crude extract was centrifuged at 8,000 rpm for 20 min, and the supernatant liquor was collected. Subsequently, the crude enzyme was purified using Ni^2+^ affinity chromatography through a HisTrap HP column (GE Healthcare, Waukesha, United States). The purified enzyme was preserved and used for subsequent assays. Protein immunoblots were performed as previously described (Wang et al., 2011). PsGH7a and the mutant were assessed using the anti-6xHis-tag primary antibody (Abclonal, College Park, MD, United States). Purified PsGH7a proteins are diluted into 100 nM for infiltration, as described by Ma (Ma Z. et al., 2015).

### Detection of Enzyme Active

The 3,5-dinitrosalicylic acid (DNS) method was employed for evaluating the cellulase activity using barley β-D-glucan and Avicel (Sigma-Aldrich) as the substrates (Song et al., 2016), respectively. The reaction system contained 150 μL of 0.2% (w/v) β-D-glucan or 1% (w/v) Avicel and 15 μg of the purified enzyme in a 300 μL reaction mixture. The hydrolysis reaction was performed at optimal 60°C and pH 4.0 for 30 min, and then terminated by adding 300 μL of DNS solution in a boiling water bath for 10 min. After the sample was cooled down to the room temperature, the absorbance was measured at 540 nm, as described by Miller (Miller, 1959). The standard curve was quantified with D-glucose. One international unit (IU) of enzymatic activity is defined as the amount of enzyme capable of releasing 1 μmol of reducing sugars per minute (Hua et al., 2018). Each experiment was performed in triplicate.

### *Phytophthora* Infection Assay on *N. benthamiana*

*Nicotiana benthamiana* leaves were harvested after infiltration and maintained on wet filter paper in Petri dishes. Infiltrated regions were inoculated with hyphal agar plugs (diameter 5 mm) of *P. sojae* and *P. capsici*, as previously described (Yang et al., 2017). The expanding lesions were photographed at 36 h after inoculation. Three independent biological replicates were included.

### CRISPR/Cas9 Knockout PsCBH7a

The *PsGH7a* gene sequence was introduced into the website^3^, and at least two sgRNA sequences were obtained by screening the specificity of the sgRNA sequence and self-circulation. The sgRNA was constructed in the pYF515 vector using the double digestion method with *Nhe* I and *Bsa* I as restriction sites. The *PsGH7a* gene was located on the FungiDB website, and upstream 1000 bp sequence and downstream 1000 bp sequence of *PsGH7a* in the whole genome of strain P6497 were found. The upstream 1000 bp and downstream 1000 bp fragment of PsGH7a was connected to the pBluescript II KS + vector, and the *mCherry* gene was inserted between them. The plasmids pYF515 and pBluescript II SK + were co-transformed into protoplast of *P. sojae* via Polyethylene-Glycol (PEG)-mediated transformation (Hua et al., 2008). After G418 resistance screening, gDNA was extracted from the transformants. The *PsGH7a* gene was trying to be amplified from gDNA of the transformants using *PsGH7a*-specific primers, and those without detected amplicons were selected for subsequent assays. The truncated fragments were sequenced to identify positive transformants.

### Pathogenicity Assay

Pathogenicity of the transformants was tested by hyphal inoculation. Hyphal agar plugs were inoculated on hypocotyls of potted soybean seedlings or leaves of soybean cultivar Williams, which is compatible with *P. sojae* strain P6497. Soybeans leaves from the second-leaf stage were used for leaf infection while the hypocotyls for hypocotyl infection. Then, the hyphal plugs were inoculated on hypocotyls or leaves and incubated at 25°C in the dark for 2 days before sampling. Place the leaves of the inoculated hypha pieces in a wet filter paper dish in the dark at 25°C for 3 days. Pictures were taken and relative virulence was measured by qRT-PCR. The ratios of *P. sojae* DNA to soybean DNA were quantified in the infected plants tissues. All these assays were repeated independently at least three times.

## Results

### Identification and Phylogenetic Analysis of *PsGH7a*

Seven candidate genes encoding GH7 cellulases (*PsGH7a* to *PsGH7g*) were identified in the *P. sojae* (strain P6497) genome. According to the predictions on SignalP Sever-5.0 (see footnote 2), four of these seven proteins (PsGH7a to PsGH7d) contain potential signal peptide, indicating those are secreted enzymes (Supplementary Table S1).

The *PsGH7a* gene is 1395 bp long with no introns (NCBI Gene ID: 20663650), encodes a 464-aa GH family 7 protein (NCBI Reference Sequence: XP_009531399.1). The expression pattern was investigated based on global digital gene expression profiling as reported (Ye et al., 2011). *PsGH7a* was highly expressed during the cyst germination and infection stages (Supplementary Table S1), and the expression pattern during infection was confirmed via quantitative reverse transcription (qRT)-PCR (Supplementary Figure S1). This indicates that *PsGH7a* may be required during *P. sojae* invasion and infection.

**FIGURE 1:**
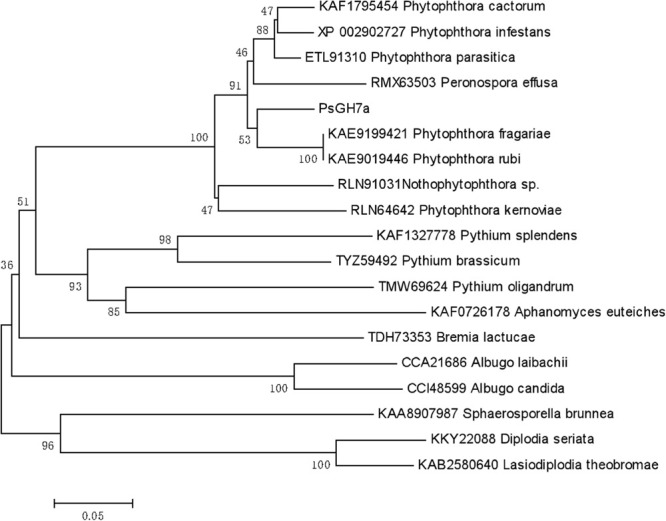
Phylogenetic tree of PsGH7a and its homologous enzymes from oomycetes and fungi. The phylogenetic tree was constructed through the MEGA 6.0 program with a neighbor-joining algorithm using 1,000 bootstrap replicates.

Based on Protein BLAST (Basic Local Alignment Search Tool)^4^ searches, the homologs of PsGH7a in some oomycetes and some other pathogen species were identified. The phylogenetic tree was constructed using PsGH7a protein sequence and the homologs from oomycetes and fungi (Figure 1). The result showed that PsGH7a-homologous proteins are widespread among plant pathogenic oomycetes and fungi. The NCBI-blast results revealed PsGH7a shares a high degree of identity (>85%) within *Phytophthora* genus (Supplementary Figure S2). Of these, PsGH7a shared 90.7% identity with the *Phytophthora fragariae* GH7 cellobiohydrolase (KAE9199421), 90.5% identity with the *Phytophthora infestans* GH7 family cellobiohydrolase (XP_002902727) and 90.0% identity with the *Phytophthora parasitica* GH7 family protein (ETL91310). Also, PsGH7a shared 86.97% identity with the GH7 family protein (RMX63503.1) of *Peronospora effusa*, which is an obligate downy mildew pathogen. That indicates PsGH7a may contribute to the biotrophic phase of pathogens.

### Biochemical Characterization of PsGH7a

**FIGURE 2:**
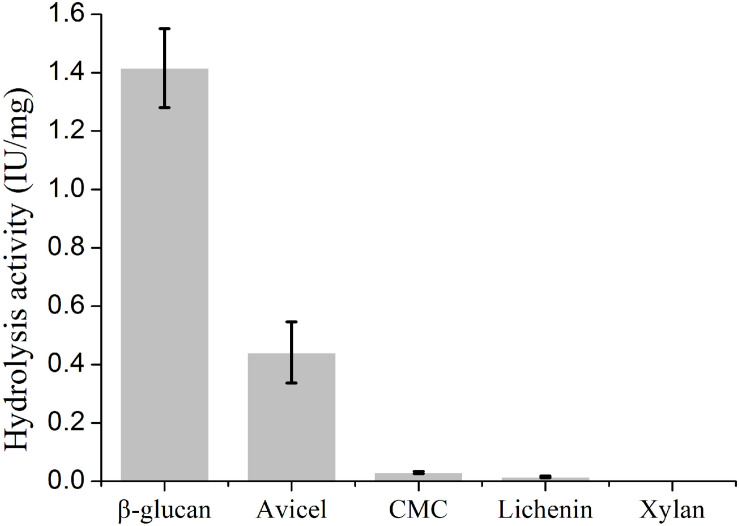
Substrate specific activity of cellobiohydrolase PsGH7a. Values are means ± SD of three replications.

To determine the biochemical properties, mature PsGH7a was heterologously expressed in *Pichia pastoris* and purified following a previously described protocol (Ma Y. A. et al., 2015). Western blotting exhibited that the purified PsGH7a protein appeared as a single band with an approximate molecular weight of 48 kDa (Supplementary Figure S3). The recombinant PsGH7a is able to efficiently hydrolyze the natural cellulose material of β-1,4-glucan (Figure 2). Evidently, PsGH7a is able to catalyze the hydrolysis of avicel, which is a typical characteristic of cellobiohydrolase (Figure 2). However, PsGH7a could not efficiently hydrolyze carboxymethyl cellulose (CMC) (Figure 2) and this phenomenon is also detected by other previous reports on cellobiohydrolases (Baramee et al., 2017; Han et al., 2018). Lichenin also could not be efficiently hydrolyzed (Figure 2), because it is composed of glucose units by the main β-1,3-1,4-glycosidic bonds which is not the preference for a β-1,4-cellobiohydrolase. Although previous reports have demonstrated that some glycoside hydrolases are bifunctional cellulase-xylanase enzymes (Hua et al., 2018; Lee et al., 2018; Chu et al., 2019), PsGH7a has no catalytic ability on the hydrolysis of xylan (Figure 2).

### PsGH7a Is an Elicitor and Induces Hypersensitive Responses in Various Plants

**FIGURE 3:**
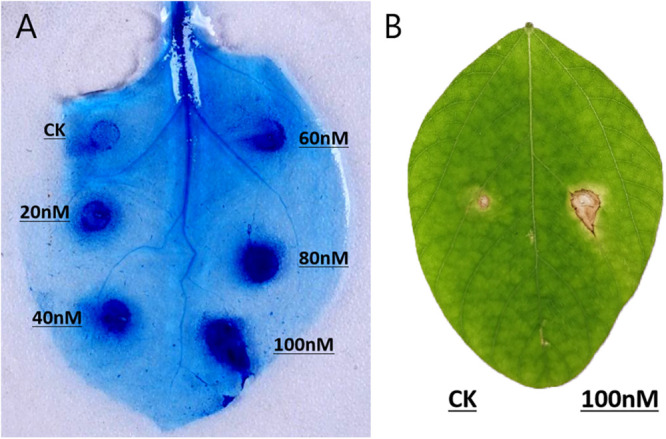
PsGH7a induces hypersensitive responses in various plants. **(A)** Protein solutions (20, 40, 60, 80, and 100 nM) and use buffer as control (CK) were infiltrated in *N. benthamiana* leaves, and then stained with trypan blue at 5 days post infiltration. **(B)** Soybean leaves were infiltrated with 100 nM protein solution and control (CK), and pictures were taken after 5 days.

The recombinant PsGH7a protein was infiltrated into expanded leaves of *N. benthamiana*. 5 days later, trypan-blue staining indicates the areas of cell death, which were enlarged with increasing concentrations of PsGH7a protein from 20 to 100 nM (Figure 3A). Compared to the other reported fungal and oomycete cellulase elicitors, the response in *N. benthamiana* is weakened and restricted to the infiltration site (Ma Y. A. et al., 2015; Ma Z. et al., 2015; Gui et al., 2017). To examine the host specificity, purified PsGH7a (100 nM) was infiltrated into expanded leaves of soybean (*Glycine max*) and obvious symptoms appeared at 5 days after infiltration (Figure 3B). Thus, PsGH7a protein can elicits hypersensitive response in the host, soybean.

### PsGH7a Promotes the Invasion of *Phytophthora*

**FIGURE 4:**
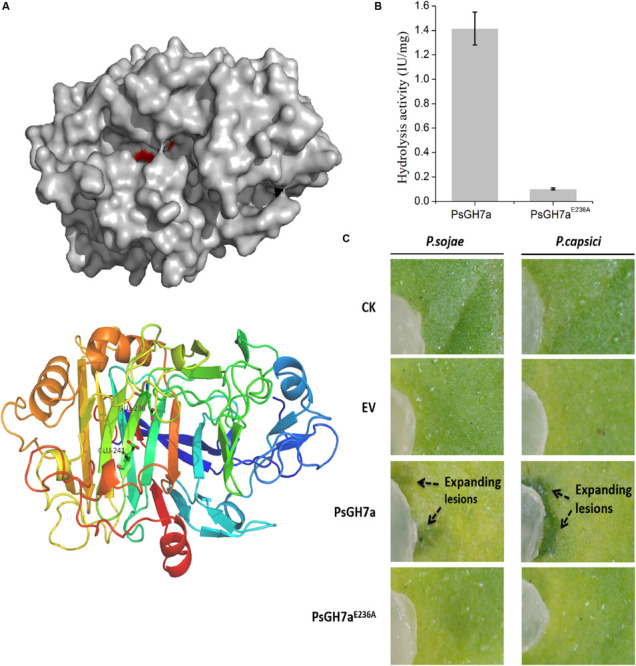
The hydrolysis of PsGH7a promotes *Phytophthora* invasion. **(A)** Predicted three dimensional structure of the PsGH7a protein obtained using the homologous *Phanerochaete chrysosporium* cellobiohydrolase Cel7D as the template (PDB: 1z3t). The color gradient shows the sequence from the N terminus (blue) to the C terminus (red). In particular, the putative catalytic residues Glu236 and Glu241 are presented as green sticks. **(B)** Cellulase activity (IU) of the wild-type and the mutant form PsGH7a^*E*236*A*^. One unit (U) of cellulase activity was defined as the amount of cellulase that catalyzed the liberation of reducing sugar equivalent to 1.0 μg glucose/min under assay conditions. Three independent biological replicates were used for each protein. **(C)** After the PsGH7a and the mutant form PsGH7a^*E*236*A*^ proteins were infiltrated into *N. benthamiana* leaves, *P. sojae* and *P. capsici* hyphal plugs were put on the infiltration sites. The buffer was infiltrated as a control (CK); the cell-free supernatant from *Pichia pastoris* GS115 which contains a empty vector (EV) was also infiltrated as another control. Arrow indicates enlarged lesion area. The pictures were taken at 3 days after inoculation.

To investigate the function of PsGH7a, a malfunction mutation was constructed. According to the structure determination of homologous GH7 cellobiohydrolases, the highly conserved residue Glu236 served as a catalytic acid in the glycosyl group hydrolysis and the conserved residue Glu241 acted as the acid/base for increasing the nucleophilicity of the catalytic water (Figure 4A and Supplementary Figure S2; Momeni et al., 2013). When the catalytic residue Glu236 was substituted with Ala, the intrinsic functional hydrogen bond in carboxyl of Glu236 was eliminated and could not effectively induce the nucleophilic attack on the anomeric carbon atom to cleave the glucosidic bond through a covalent glycosyl-enzyme intermediate (Davies et al., 1993; Vocadlo and Davies, 2008; Supplementary Figure S4). Besides, in mutant PsGH7a^*E*236*A*^, the extended distance between Ala236 and Glu241 is adverse to the substrate binding, and this single mutation was not sufficient to induce comformational change of the active site architecture. Therefore, the mutant on the Glu236 (PsGH7A^*E*236*A*^) did not affect the stability and molecular mass of PsGH7a, and can not induce cell death at 5 dpi (Supplementary Figure S3).

The catalytic activity assay indicates that PsGH7a^*E*236*A*^ showed much lower activity than wild type (Figure 4B). The purified PsGH7a and PsGH7a^*E*236*A*^ was infiltrated into *N. benthamiana* leaves, and hyphal plugs of *P. sojae* and *P. capsici* were put on the infiltration sites. Three days after inoculation, the lesions on the area with PsGH7a were significantly larger than others on the leaves (Figure 4C). The results indicated that the infiltration of PsGH7a in leaves increased their susceptibility to *P. sojae* and *P. capsici*, and its enzymic activity is also required.

### CRISPR/Cas9 Genome Editing for PsGH7a Knockout

**FIGURE 5:**
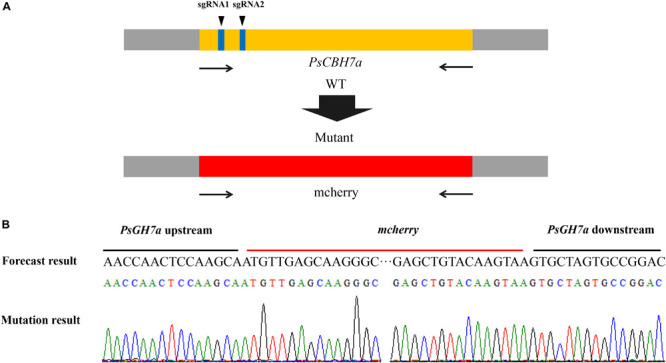
CRISPR/Cas9 Genome Editing for PsGH7a Knockout. **(A)** sgRNA1, sgRNA2 are designed to target PsGH7a, and the mCherry gene with PsGH7a flanking sequences are used as template for fragment homologous substitution. **(B)** The sequencing results consist with the forecast result, indicates the PsGH7a gene have been successfully replaced as mcherry. The DNA used for sequencing are obtained from transformants, and the sequencing peaks are attached.

The contribution of PsGH7a to *P. sojae* virulence was investigated through CRISPR/Cas9 genome editing. The *PsGH7a*-knockout mutants of *P. sojae* (strain P6497) via CRISPR/Cas9 system were generated following a previously described protocol (Fang and Tyler, 2016). Two single guide RNAs (sgRNAs) with independent targeting were designed to disrupt the *PsGH7a* coding region (Figure 5A). The plasmid pBS KS + containing a homologous donor DNA (the *mCherry* gene with *PsGH7a* flanking sequences are used as a template for fragment homologous substitution, Figure 5A) and pYF515 vectors carrying each sgRNA information and *hSpCas9* sequence were introduced into *P. sojae* via protoplast transformation as described (Hua et al., 2008). Six independent transformants were identified by gDNA PCR (Supplementary Figure S5) and sequencing screening, which all showed normal filamentous growth. The sequencing result showed that the *PsGH7a* gene was replaced by inserted *mCherry* in the mutants TG1 and TG6 (Figure 5B). The transformant TG3 is failed to acquire *PsGH7a* deletion and be used as the assay control (CK) (Supplementary Figure S5).

### PsGH7a Is Required for Full Virulence of *P. sojae*

**FIGURE 6:**
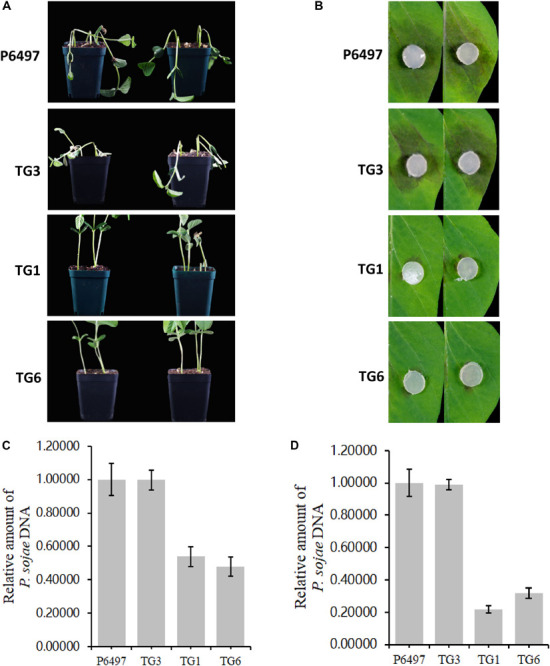
PsGH7a is required for full virulence of *P. sojae*. **(A)** P6497 wild-type and transformants hyphae plugs infected wounded hypocotyls, the pictures were taken at 3 days later. **(B)** P6497 and transformants hyphae plugs infected soybean leaves, the pictures were taken at 3 days later. **(C)** Determination of *P. sojae* DNA content in infected hypocotyls. Hypocotyls were collected at 3 days after infection with P6497 and transformants, and DNA was extracted for real-time PCR. **(D)** Determination of *P. sojae* DNA content in leaves. Leaves were collected 3 days after infection with P6497 and transformants, and DNA was extracted for real-time PCR. Three independent replicates of each real-time PCR.

Potted seedlings of the soybean susceptible cultivar (Williams, without any known resistance genes) were inoculated with hyphal plugs on the wounded hypocotyls, respectively. After 4 days, the recipient strain P6497 killed soybean seedlings quickly while the transformants did not (Figure 6A). Consistent with these results, the virulence of these transformants was damnified on soybean leaves also. Hyphal agar plugs were inoculated on the opposite leaves of Williams, and moisturized in petri dishes for 2 days. The lesion regions caused by TG1 and TG6 were much smaller than WT and CK (Figure 6B). The biomass quantification showed the relative amount of *P. sojae* DNA of the mutants were significantly reduced in the inoculated soybean hypocotyls and leaves compared to WT and CK (Figures 6C,D). These results suggest that PsGH7a is required for full virulence of *P. sojae* in soybean infection.

## Discussion

Plants and pathogens are involved in a dynamic co-evolutionary struggle for survival. To cope with pathogen infection, plants rely on the physical barriers and layered innate immunity to program defenses (Jones and Dangl, 2006; Wang et al., 2019). Plant cell walls provide the primary physical defense, and may be dynamic strengthened for added defense during interaction with pathogens (Vorwerk et al., 2004). To penetrate plant cell walls and colonization of living host tissue, phytopathogens deploy secreted CWDEs as part of their arsenals (Hématy et al., 2009). The enzymes include glucanases, cellulases, polygalacturonases, pectinases and xyloglucanases (Vorwerk et al., 2004). For example, *Ustilago maydis*, the causal agents of corn smut, contains 33 such CWDE-encoding genes, (Kamper et al., 2006). However, there is little detailed information available about oomycete apoplastic CWDEs to date (Ma et al., 2017).

The CWDEs target multiple plant polysaccharides. Here we identified a *P. sojae* GH7 family protein, PsGH7a, which is a typical CBH GH7 family has been mainly found in fungi, our results showed that GH7 proteins are conserved in oomycetes. Both phytopathogenic fungi and oomycete are able to break the intact physical surfaces of host plants, which indicates the hydrolytic activity provided by GH7 enzymes maybe employed by these pathogens. Our results showed that the PsGH7a is highly conserved in both oomycetes and fungi (Figure 1). The PsGH7a shares a high degree of identity (>85%) within *Phytophthora* genus (Supplementary Figure S2), and shared 86.97% identity with the *Peronospora effusa* GH7 family protein (RMX63503.1). That indicates GH7a of *Phytophthora* may contribute to the biotrophic phase. The homologs from saprophytic oomycetes, including *Aphanomyces euteiches*, *Pythium oligandrum*, and *Pythium brassicum*, and *Pythium splendens*, share 60–65% identity with PsGH7a, are clustered to adjacent branch. The homologs from other obligate oomycetes, such as *Bremia lactucae, Albugo candida* and *Albugo laibachii*, are clustered to sub-groups. The homologs from fungi, including *Sphaerosporella brunnea*, *Diplodia seriata*, and *Lasiodiplodia theobromae*, are clustered into sub-population, that means they are evolved independently. These differences suggest that independent evolutionary events may have occurred in target sequences of oomycetes and fungi.

In the hydrolysis activity detection assay, PsGH7a is able to hydrolyze β-1,4-glucan and avicel. The main load-bearing component of plant cell wall is cellulose, which is the β-1,4-linked homopolymer of glucosides (Vorwerk et al., 2004). Thus, β-1,4-glucan represents the major component of cellulose in plant cell wall (Glass et al., 2013). In addition, the highly ordered arrangement of cellulose fibers connected by regular hydrogen bonds is termed as the crystalliferous region of cellulose, which is recognized as the critical traffic jam for reducing hydrolytic efficiency of cellulases on cellulose surface (Igarashi et al., 2011). The crystallinity of cellulosic avicel plays a major role in determining the rate of hydrolysis by cellulases, especially for CBH (Hall et al., 2010). As CBHs attach to cellulose chain ends, immediately triggering the internal bond cleavage by EGs, CBH can immediately capture the newly exposed reducing chain ends (Kurasin and Valjamae, 2011). So PsGH7a is identified as a cellobiohydrolase, provides the majority of hydrolytic turnover.

Usually, the hydrolysis activities of CWDE cocktails are required for phytopathogen virulence, which help to macerate plant tissues during infection (King et al., 2011). Silencing of *P. sojae XEG1*, which encodes a GH12-family xyloglucanase, severely reduced virulence (Ma Z. et al., 2015). Consistent with these results, pathogenicity assays showed the *P. sojae* lose its virulence when *PsGH7a* was knocked out. These results suggest that the PsGH7a plays key roles during *P. sojae* invasion. Furthermore, infiltration of PsGH7a even promote invasion of *P. sojae* and *P. capsici* on the non-host *N. benthamiana* leaves. So far, little is known about the differences in components of cell wall between hosts and non-hosts, and also the chemical composition is a factor in the outcome of the non-host disease resistance (Somerville et al., 2004). Usually, pathogenic CWDEs have obvious selectivity for hosts and good adaptability to the most preferred host (King et al., 2011), so it is possible to control fungus and oomycete disease by fine-tuned dynamic enhancement of plant cell walls. Our progress in defining *P. sojae* GH7a has provided new insights into the possible role of cell wall composition in controlling disease interactions.

In the co-evolution of plants and pathogens, some conserved CWDEs secreted by phytopathogens are recognized as pathogen-associated molecular patterns (PAMPs) by plant cell surface pattern recognition receptors (PRRs), and trigger plant immunity (PTI) (Wang and Wang, 2018). For example, an endocellulase from *Rhizoctonia solani* is an elicitor (Ma Y. A. et al., 2015). *P. sojae* XEG1 act as virulence factors and PAMPs in oomycetes (Ma Z. et al., 2015). Two GH12 proteins VdEG1 and VdEG3 produced by the fungus *Verticillium dahliae* Vd991 acted as PAMPs to trigger cell death (Gui et al., 2017). Plant cells also have molecular mechanisms for sensing and responding to cell wall derangement. Some of the cell wall degradation fragments produced by pathogen CWEDs, which are termed damage-associated molecular patterns (DAMPs), can elicit defensive responses by plant (Hématy et al., 2009). For example, oligogalacturonic acid, which is produced when degradation of pectins by polygalacturonase generates, can trigger plant defenses (D’Ovidio et al., 2004). We found that PsGH7a protein can elicits hypersensitive response in tobacco and soybean. The necrotic lesions usually appear at 3 to 5 days post protein infiltration, it is hard to say the response is due to PAMP or DAMP. For the protein mutant PsGH7a E236A showed much lower activity than wild type, and cannot induce HR on soybean leaves, that indicate the degradation product but not the protein triggers PTI. We thought maybe the degradation product but not the protein triggers cell death, and for some reason, the cell death spread out slowly from the wound, while control or the protein mutant PsGH7a E236A does not. Maybe that is a kind of DAMP-triggered immunity. Also, we noticed that the cell death was increased with increasing concentrations of enzyme, so we guess it is probably because more damage was created by PsGH7a, which had been proved as a cellobiohydrolase.

It is well reported a novel decoy strategy is used in *Phytophthora* pathosystems (Ma et al., 2017). *P. sojae* manipulates plant immunity by protecting the GH12-family xyloglucanase XEG1 through its truncated paralogous PsXLP1 as a decoy. It is worth to notice that the CAZy database lists nearly 5000 GH7 sequences, and the GH7 cellulases in *Phytophthora* offer complicated evolutionary branches with each other. The decoy pattern provides an explanation for the multiple, similar sequences from the same family. The cross fire at the front line of plant defense is always the most intense. Thus, the researches on *Phytophthora* CWDEs provide more novel viewing angles of evolutionary struggle between plants and pathogens.

## Data Availability Statement

The datasets generated for this study can be found in the NCBI XP_009531399, NCBI KAE9199421.1, NCBI KAE9019446.1, NCBI ETL91310.1, NCBI KAF1795454.1, NCBI XP_002902727.1, NCBI RLN91031.1, NCBI RMX63503.1, NCBI RLN64642.1, NCBI KAF1327778.1, NCBI TYZ59492.1, NCBI TDH73353.1, NCBI CCA21686.1, NCBI KAF0726178.1, NCBI CCI48599.1, NCBI KAA8907987.1, NCBI KKY22088.1, and NCBI KAB2580640.1.

## Author Contributions

QW and CH designed the experiments. YH, XT, and CH wrote the manuscript, and performed the experiments and data analysis. YH and CH expressed the proteins and test the catalytic activity. YH tested the virulence of *Phytophthora*. XT constructed the *P. sojae* mutants. QX, YJ, and XH participated in manuscript revision or experiment. QW revised the manuscript and provided the funding for this research. All authors contributed to the article and approved the submitted version.

## Conflict of Interest

The authors declare that the research was conducted in the absence of any commercial or financial relationships that could be construed as a potential conflict of interest.

## Footnotes

1 http://www.cazy.org

2 http://www.cbs.dtu.dk/services/SignalP/

3 http://grna.ctegd.uga.edu/

4 https://blast.ncbi.nlm.nih.gov/Blast.cgi
