# Supplementary material for: A Conserved Glycoside Hydrolase Family 7 Cellobiohydrolase PsGH7a of Phytophthora sojae Is Required for Full Virulence on Soybean
Source: Front Microbiol. 2020 Jul 2;11:1285. doi: 10.3389/fmicb.2020.01285 (PMC7343703; doi:10.3389/fmicb.2020.01285)
Supplement: Supplementary file 1 [file Presentation_1.pptx]

## Slide 1
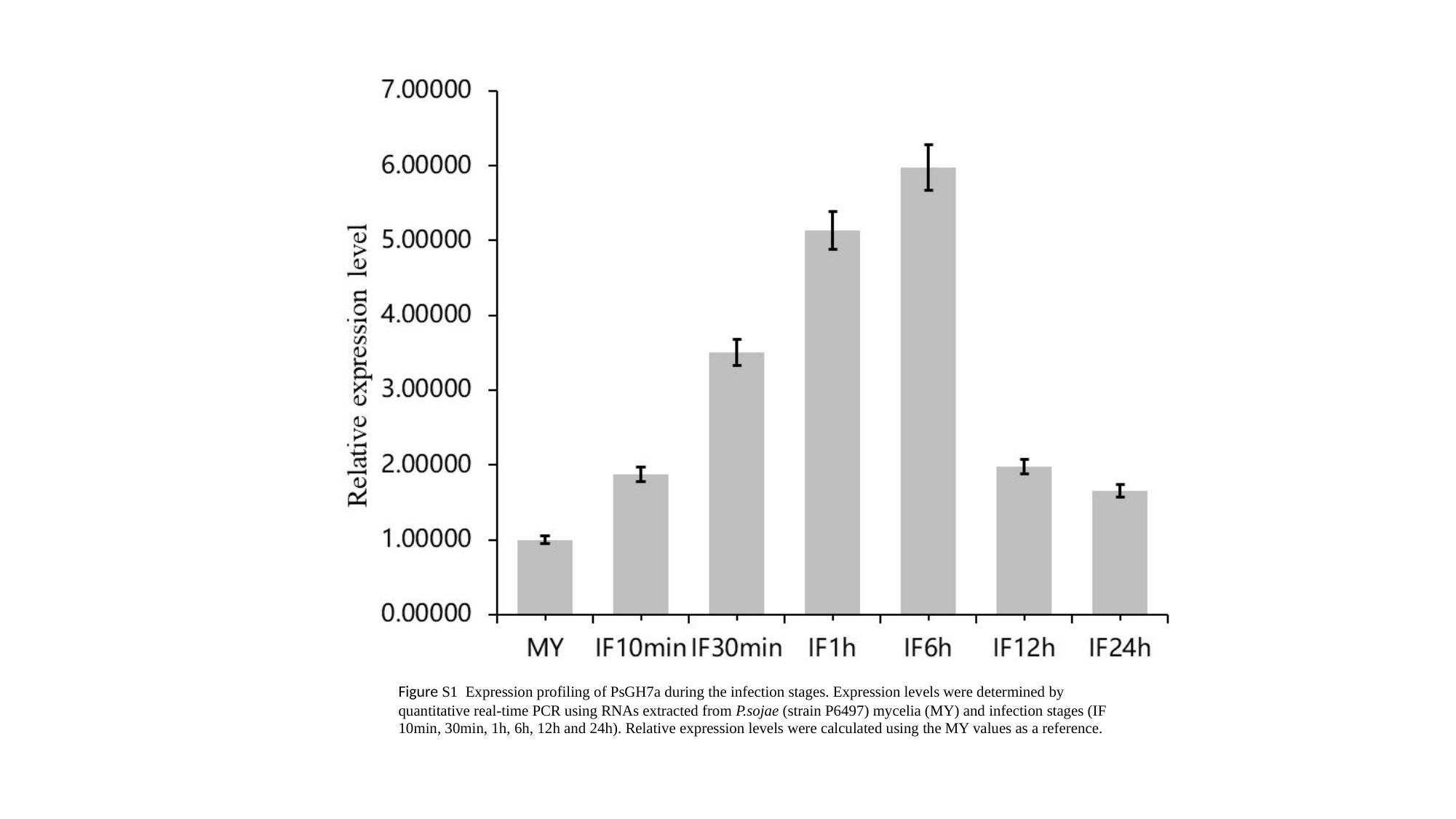

Figure S1 Expression profiling of PsGH7a during the infection stages. Expression levels were determined by quantitative real-time PCR using RNAs extracted from P.sojae (strain P6497) mycelia (MY) and infection stages (IF 10min, 30min, 1h, 6h, 12h and 24h). Relative expression levels were calculated using the MY values as a reference.

## Slide 2
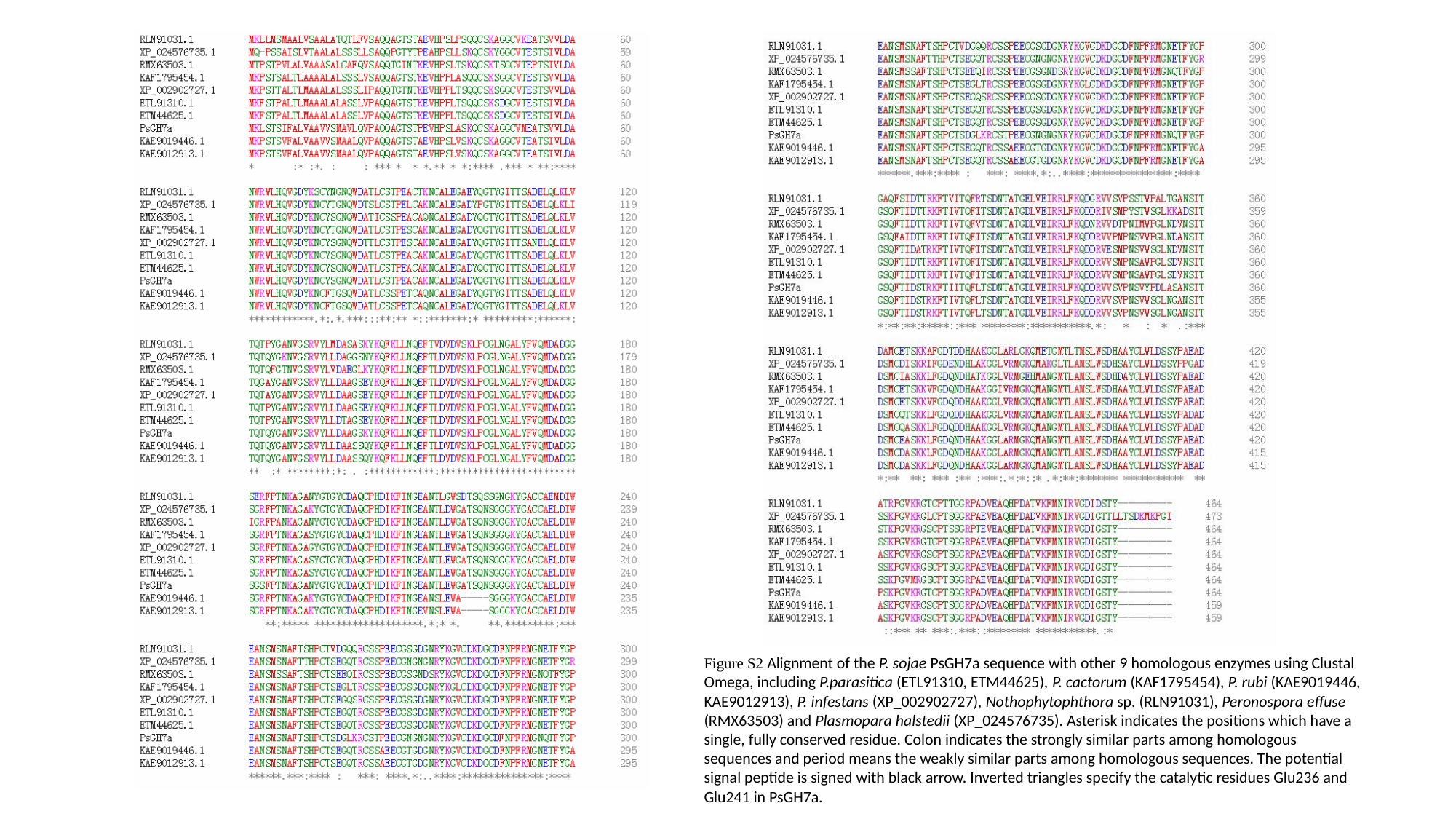

Figure S2 Alignment of the P. sojae PsGH7a sequence with other 9 homologous enzymes using Clustal Omega, including P.parasitica (ETL91310, ETM44625), P. cactorum (KAF1795454), P. rubi (KAE9019446, KAE9012913), P. infestans (XP_002902727), Nothophytophthora sp. (RLN91031), Peronospora effuse (RMX63503) and Plasmopara halstedii (XP_024576735). Asterisk indicates the positions which have a single, fully conserved residue. Colon indicates the strongly similar parts among homologous sequences and period means the weakly similar parts among homologous sequences. The potential signal peptide is signed with black arrow. Inverted triangles specify the catalytic residues Glu236 and Glu241 in PsGH7a.

## Slide 3
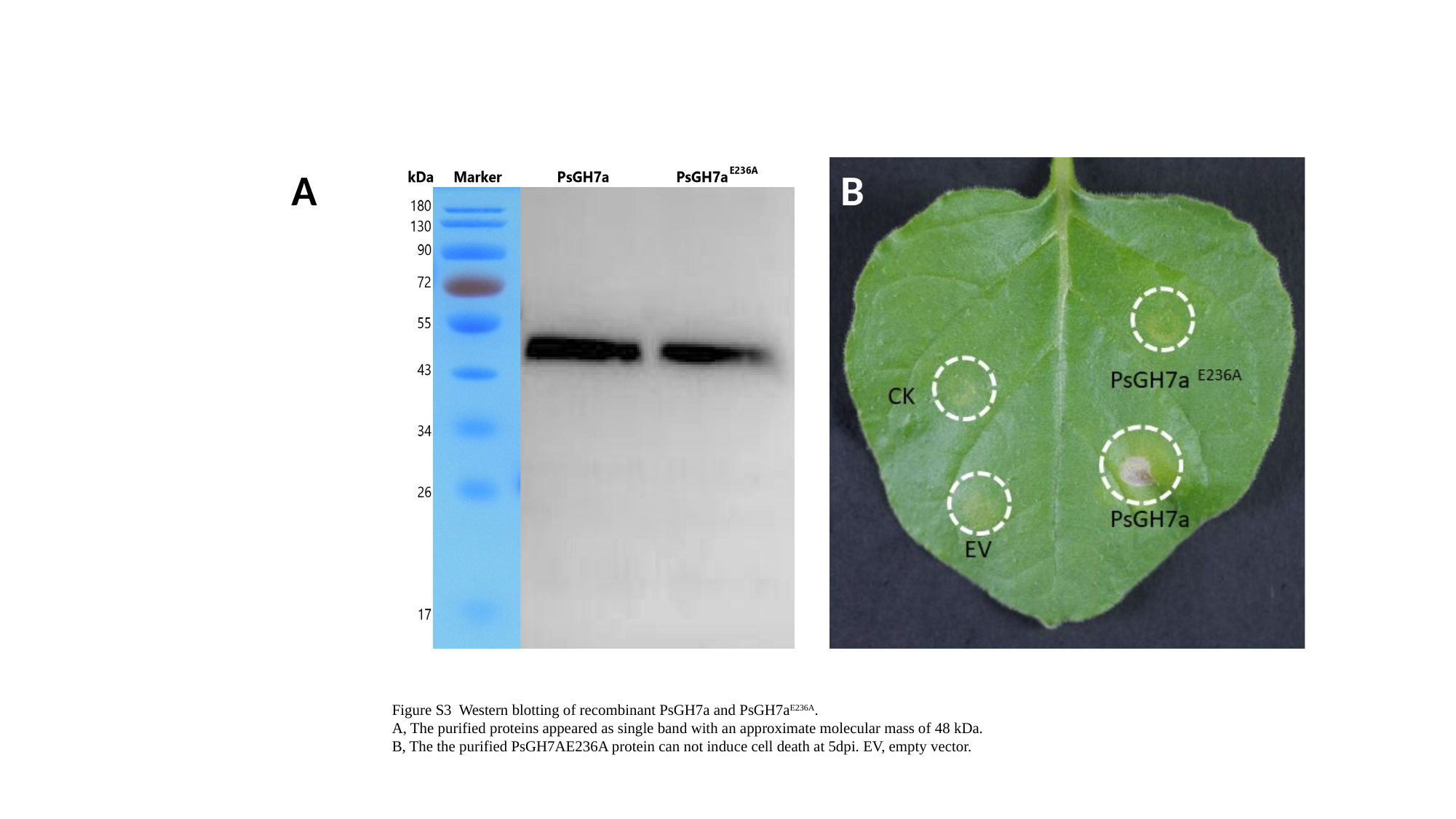

A
B
Figure S3 Western blotting of recombinant PsGH7a and PsGH7aE236A. A, The purified proteins appeared as single band with an approximate molecular mass of 48 kDa.
B, The the purified PsGH7AE236A protein can not induce cell death at 5dpi. EV, empty vector.

## Slide 4
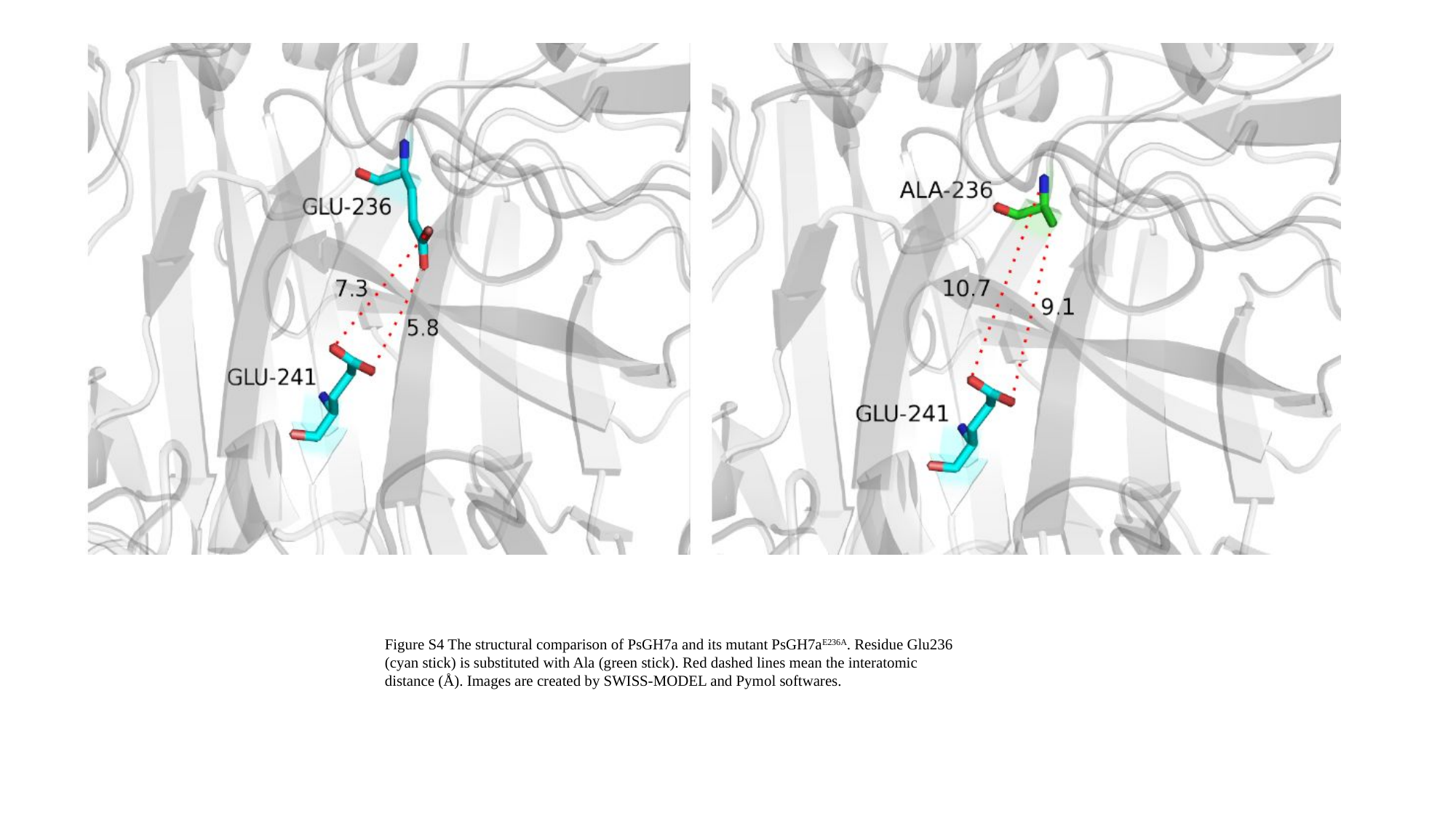

Figure S4 The structural comparison of PsGH7a and its mutant PsGH7aE236A. Residue Glu236 (cyan stick) is substituted with Ala (green stick). Red dashed lines mean the interatomic distance (Å). Images are created by SWISS-MODEL and Pymol softwares.

## Slide 5
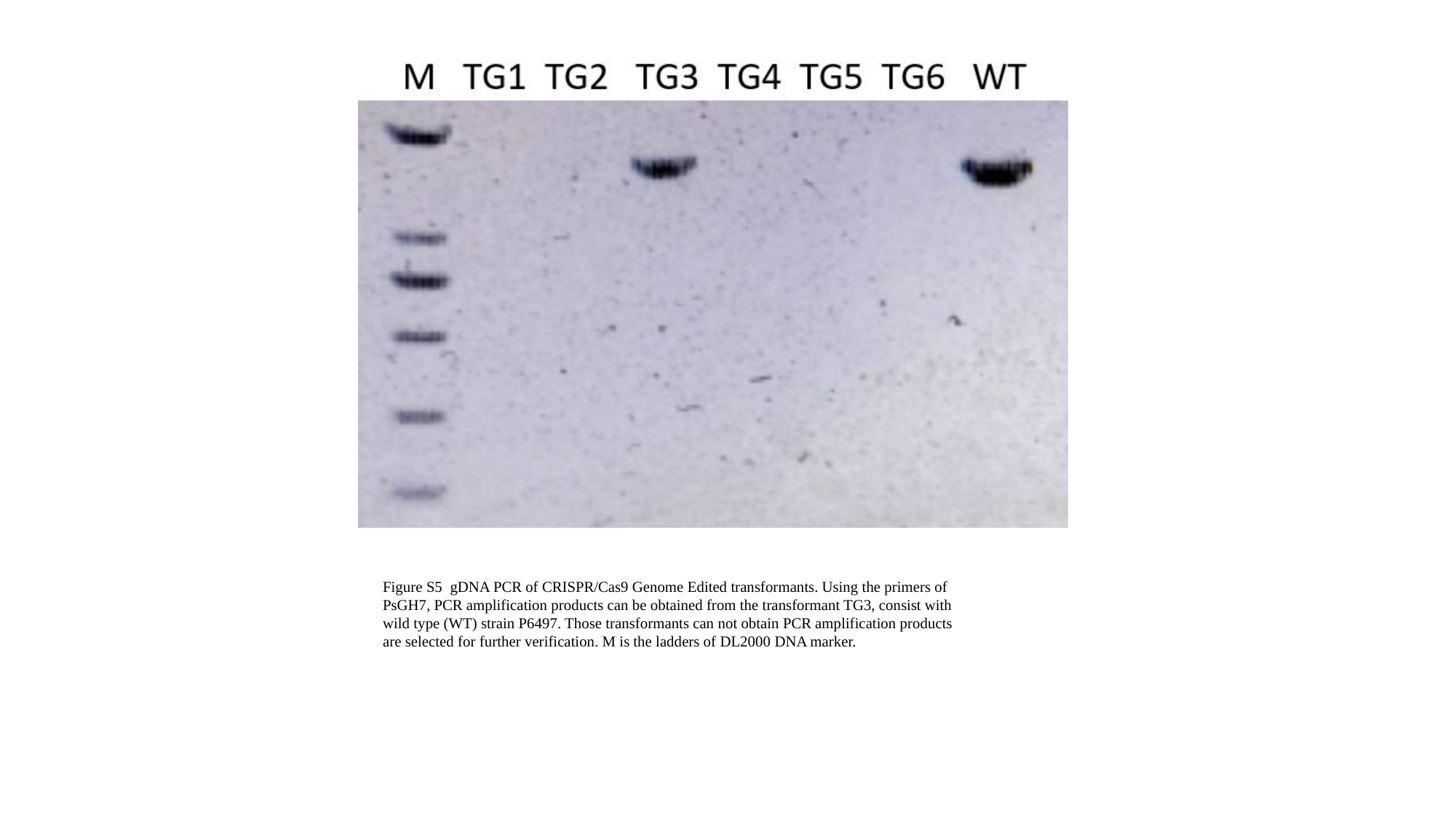

Figure S5 gDNA PCR of CRISPR/Cas9 Genome Edited transformants. Using the primers of PsGH7, PCR amplification products can be obtained from the transformant TG3, consist with wild type (WT) strain P6497. Those transformants can not obtain PCR amplification products are selected for further verification. M is the ladders of DL2000 DNA marker.
